# Supplementary material for: A high-resolution mRNA expression time course of embryonic development in zebrafish
Source: eLife. 2017 Nov 16;6:e30860. doi: 10.7554/eLife.30860 (PMC5690287; doi:10.7554/eLife.30860)
Supplement: Supplementary file 6. [file elife-30860-supp6.zip › biolayout-clusters-files/Cluster190-genes.html]

Cluster190


# Cluster190: Genes

| Ensembl ID | Gene Name | Chr | Start | End | Biotype |
| --- | --- | --- | --- | --- | --- |
| ENSDARG00000002255 | epb41l3a | 24 | 42055974 | 42102897 | protein\_coding |
| ENSDARG00000075132 | fh | 12 | 47638965 | 47650718 | protein\_coding |
| ENSDARG00000039093 | got1 | 13 | 40311660 | 40331103 | protein\_coding |
| ENSDARG00000017772 | mdh1aa | 1 | 50325109 | 50347087 | protein\_coding |
| ENSDARG00000043371 | mdh2 | 10 | 33439147 | 33448309 | protein\_coding |
| ENSDARG00000103095 | tbk1 | 4 | 120072 | 134990 | protein\_coding |
| ENSDARG00000019033 | tmem59 | 2 | 26956940 | 26965271 | protein\_coding |
